# Pure laparoscopic liver resection for giant liver hemangioma with extrahepatic growth based on preoperative 3-dimensional simulation: A case report

**DOI:** 10.1186/s40792-019-0607-8

**Published:** 2019-04-01

**Authors:** Yuichiro Okumura, Takehiro Noda, Hidetoshi Eguchi, Takehiko Hanaki, Yoshifumi Iwagami, Hirofumi Akita, Tadafumi Asaoka, Kunihito Gotoh, Shogo Kobayashi, Koji Umeshita, Masaki Mori, Yuichiro Doki

**Affiliations:** 1 Department of Gastroenterological Surgery, Graduate School of Medicine, Osaka University, 2-2, Yamadaoka E-2, Suita, Osaka 565-0871 Japan; 2 Division of Health Science, Graduate School of Medicine, Osaka University, Osaka, Japan; 3 Department of Surgery and Science, Graduate School of Medical Sciences, Kyushu University, Fukuoka, Japan

**Keywords:** Giant hemangioma, Laparoscopic liver resection, 3D simulation

## Abstract

**Background:**

Performing laparoscopic liver resection for giant hemangiomas is challenging, and careful preoperative planning is essential. Controlling intraoperative bleeding and handling surgical instruments within a limited workspace is necessary.

**Case presentation:**

In the present case, the patient was a 38-year-old woman diagnosed with a 16-cm giant liver hemangioma in segment 5/6, with extrahepatic growth. Preoperative three-dimensional simulations for port placement and the laparoscopic view from the left upper abdomen were performed to complete the pure laparoscopic liver resection. The laparoscopic resection was then safely performed on the same way.

**Conclusions:**

Pure laparoscopic resection could be applied to giant hemangiomas with extrahepatic growth, and the preoperative three-dimensional simulation of port placement and the laparoscopic view might be helpful when the intraabdominal workplace is restricted.

**Electronic supplementary material:**

The online version of this article (10.1186/s40792-019-0607-8) contains supplementary material, which is available to authorized users.

## Introduction

Cavernous hemangiomas are the most common type of benign liver tumors, and the majority of cavernous hemangiomas are incidentally detected by imaging studies performed for other reasons [1]. A recent national survey reported that liver hemangiomas measuring > 10 cm would be candidates for surgery in patients with symptoms [2]. Compared with open liver resection, laparoscopic liver resection (LLR) has many advantages including shorter hospital stay, less blood loss, and earlier postoperative recovery [3]. The current indication for LLRs is a solitary tumor with a ≤ 5-cm diameter [4]. LLR for giant hemangiomas > 10 cm is challenging due to insufficient space for manipulation and bleeding risk [2, 3]. Here, we present a case with a symptomatic giant hemangioma 16 cm in diameter that was successfully resected by LLR after utilizing three-dimensional (3D) simulation technology to preoperatively plan port placement and the laparoscopic view.

## Case presentation

A young woman was referred to our hospital for a hepatic mass, which presented with epigastric pain. The patient had no notable past medical history. She did not smoke or abuse alcohol. Her physical examination revealed slight epigastric tenderness. Contrast-enhanced computed tomography (CT) demonstrated a 9-cm mass in segments 5/6 of the liver, with extrahepatic growth. The radiological findings were compatible with cavernous hemangioma. Because the patient’s symptoms soon disappeared, she decided to receive surveillance. For 6 years after her first visit to the hospital, the hemangioma grew slowly. However, during the seventh year, the tumor grew from 13 cm to 16 cm, and surgery was indicated.

**Fig. 1:**
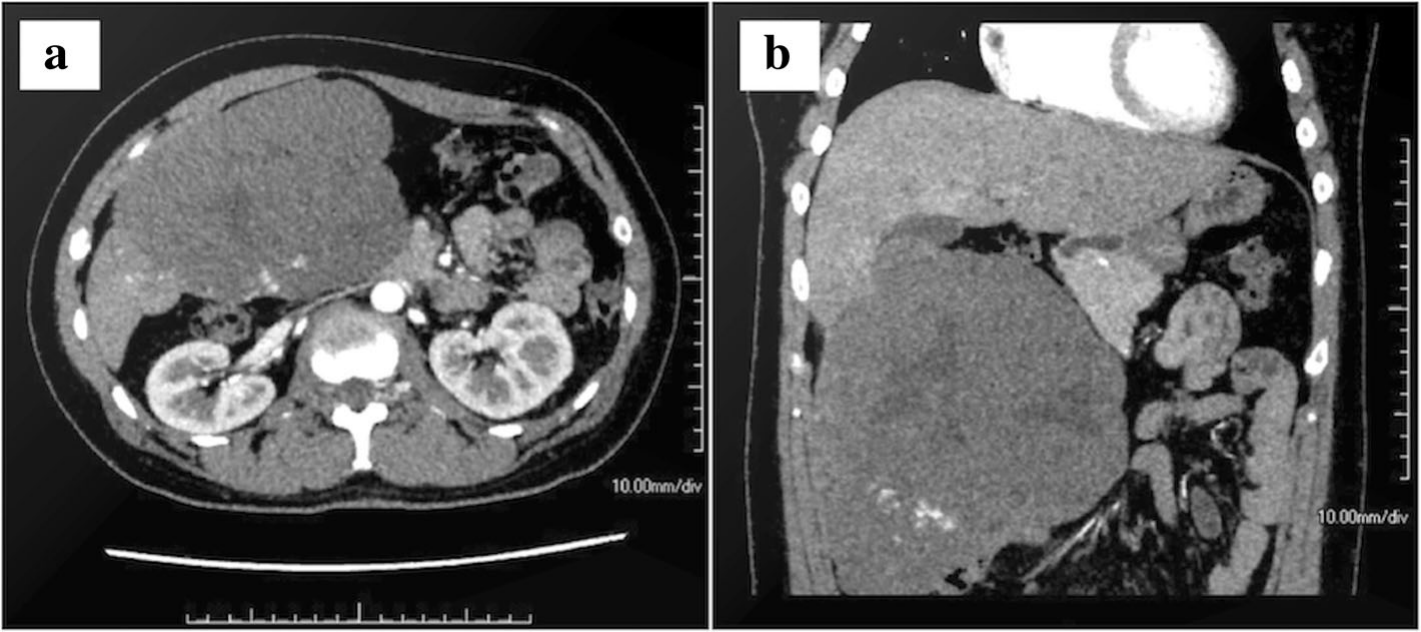
Contrast-enhanced computerized tomography showing a giant liver hemangioma originating from segments 5/6, with extrahepatic growth. **a** Axial section. **b** Coronal section

**Fig. 2:**
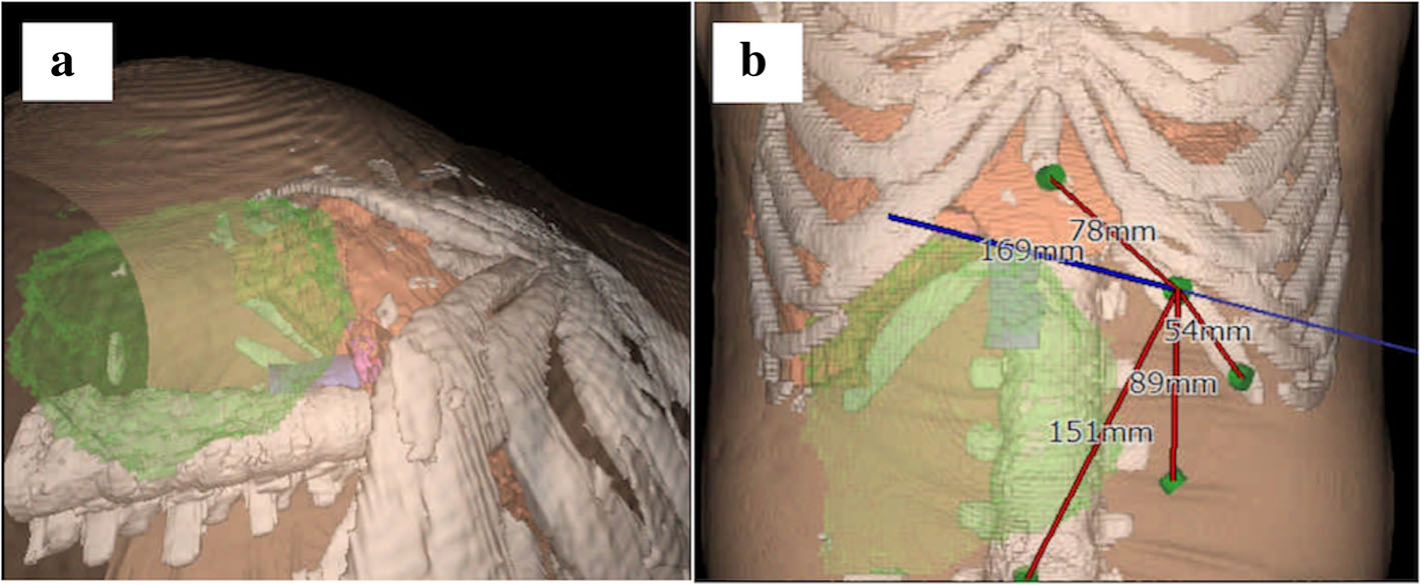
Preoperative three-dimensional simulation images. **a** Reconstructed laparoscopic view from the left upper abdomen. **b** Port placement and the minimal straight parenchymal cutline were simulated


Preoperative laboratory tests revealed that the patient’s hemoglobin level was slightly decreased at 116 g/L, but the liver function tests, platelet counts, and coagulation factors were all within the normal ranges. Figure 1a and b show the CT images of the giant hemangioma originating from segments 5/6, with a maximum diameter of 16 cm. The preoperative 3D simulation was conducted using SYNAPSE VINCENT (Fujifilm Medical, Tokyo, Japan). The laparoscopic view and parenchymal cutline for the pure laparoscopic approach were simulated by inserting trocars mainly on the left upper abdomen (Fig. 2a, b). The positional relation between the hemangioma and the vasculature was also simulated (Additional file 1: Video 1). Transcatheter arterial embolization (TAE) to A5 as a main feeder was performed using Embosphere^Ⓡ^, which was diluted 100 times, on the day before surgery.

**Additional file 1: Video 1.** The shortly edited movie of the three-dimensional simulation view from the left upper abdomen and the whole intraoperative video. (MP4 19570 kb)


**Fig. 3:**
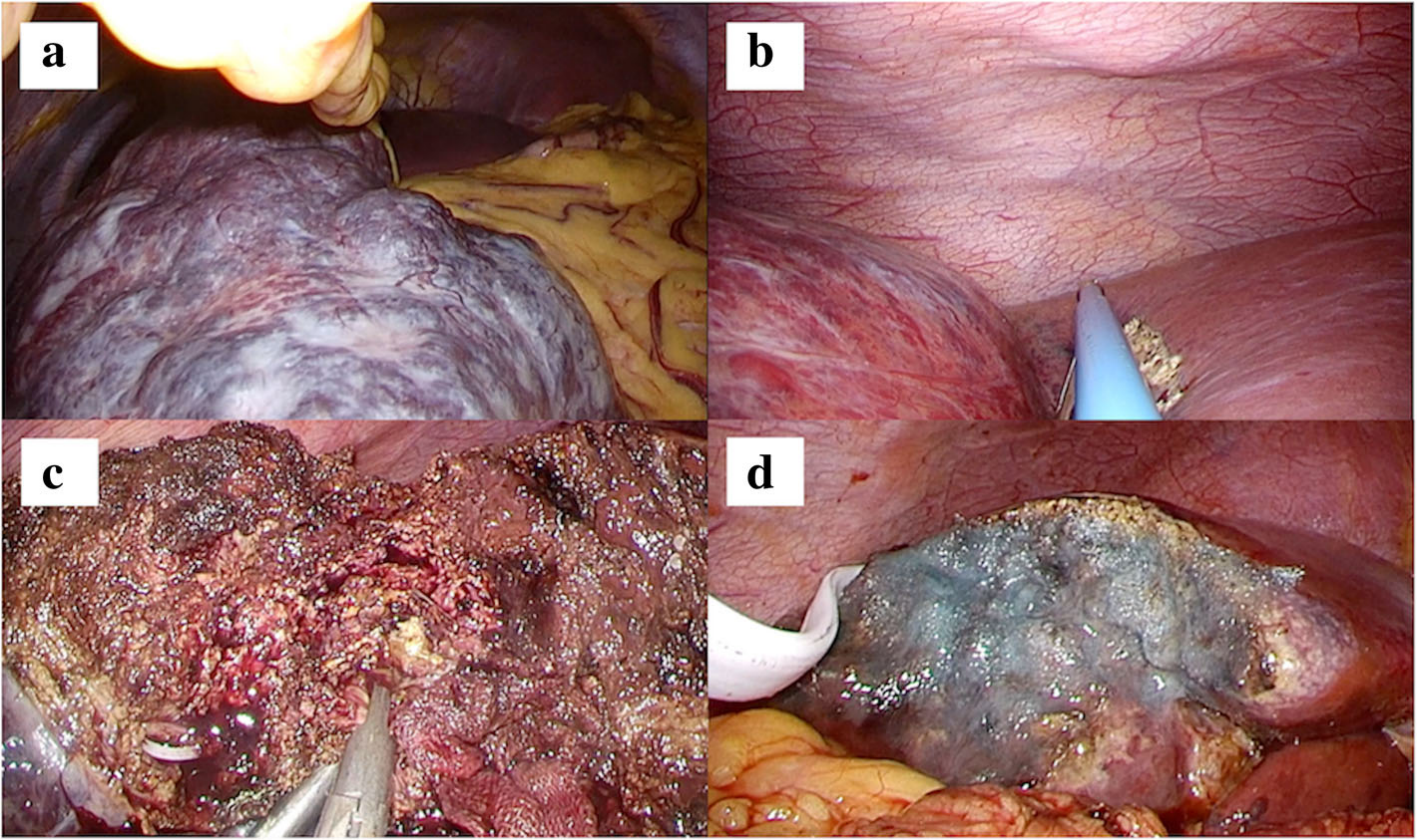
**a** The giant hemangioma occupying the entire space of the right upper abdomen was observed from the umbilical laparoscopic view. **b** A straight transection cutline was marked on the liver surface from the left upper laparoscopic view. **c** Hepatic transection. **d** After the resection, a drainage tube was placed adjacent to the resection margin

**Fig. 4:**
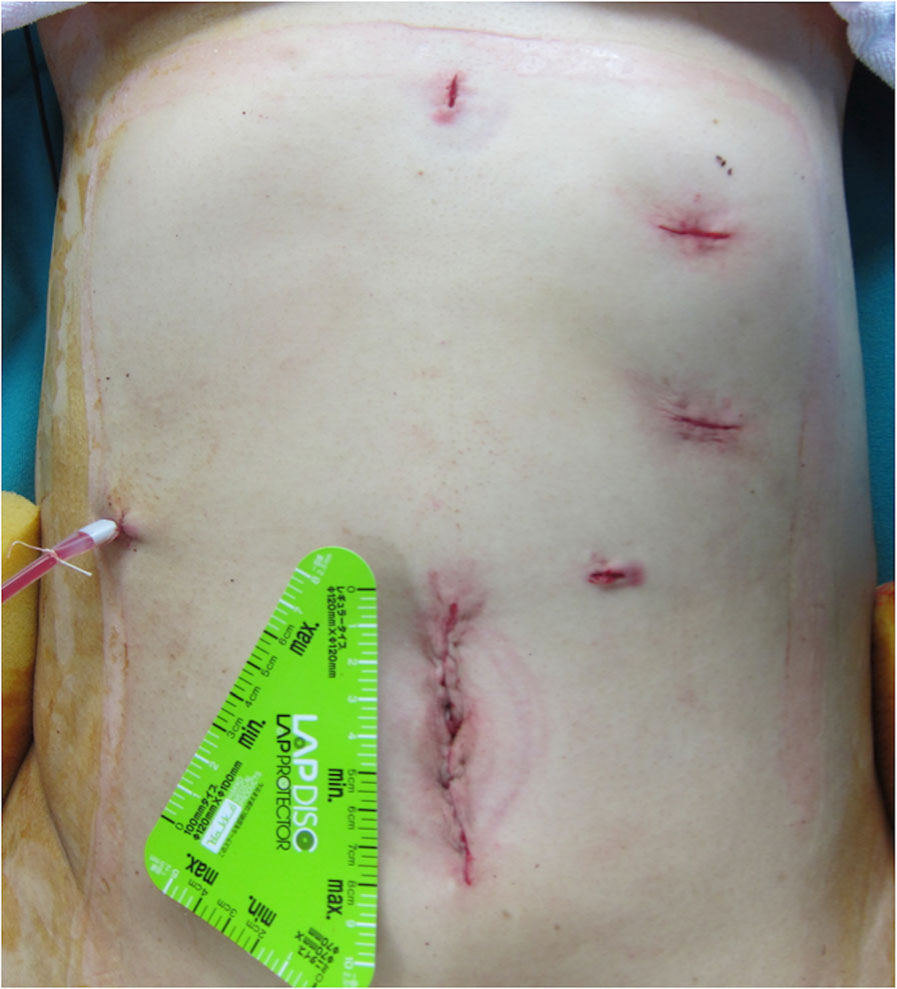
Postoperative abdominal incision. The umbilical trocar incision was slightly extended and used to extract the specimen

During laparoscopy, carbon dioxide was used for peritoneal insufflation, and the abdominal pressure was maintained between 8 and 12 mmHg. Observed from the umbilical laparoscopic view, the giant hemangioma occupied the whole space (Fig. 3a). All ports were placed as in the preoperative simulation. The hepato-duodenum ligament was encircled by approaching the lesser omentum. An umbilical tape was guided to the omental foramen, using snake retractor (Additional file 1: Video 1). Pringle maneuver was applied during the transection. The straight resection line was confirmed using intraoperative ultrasound, and hepatic transection was done by laparosonic coagulating shears (Ethicon Endo-Surgery, Cincinnati, OH, USA), an ultrasonic surgical aspirator (CUSA; Cavitron Lasersonic Corp., Stamford, CT, USA), and a bipolar clamp coagulation system (the VIO 300D; ERBE Elektromedizin, Tübingen, Germany) (Fig. 3b, c). The hemangioma was then extracted using a plastic bag through the slightly enlarged umbilical trocar incision (Fig. 4). A drainage tube was placed adjacent to the resection margin (Fig. 3d). The operative time was 288 min, and total blood loss was 200 mL (Additional file 1: Video 1). Transfusion was not required. The resected tumor was 16 × 16 × 9 cm in diameter and weighed 980 g. Histopathological findings were compatible with cavernous hemangioma. The patient had an uneventful recovery.

## Discussion

Cavernous liver hemangiomas are typically observed in adults aged 30-50 years, and occur three to five times more frequently in women [1]. Giant hemangiomas may be complicated by hematologic or coagulation system abnormalities known as Kasabach-Merritt syndrome [2, 5], and some patients complain of abdominal symptoms. The operative indication for liver hemangiomas is determined by their size, growth rate, and presence of symptoms. Prophylactic resection in asymptomatic patients solely due to size is regarded as inadequate [6]. Sakamoto et al. conducted the national survey of Japan and reported that about half of patients with 10–15-cm hemangiomas had no clinical symptoms and could be candidates for careful observation [2]. Our patient received observation, with imaging done every 6 months, but the tumor rapidly increased in size and exceeded 15 cm, fulfilling the indication for hepatic resection.

LLR was first introduced in 1992 and is increasingly used due to new technologies and devices [7]. Compared to open liver resection, LLR has several advantages including decreased blood loss, shorter hospital stay, and improved cosmetic results. The acceptable indication for LLR according to the Louisville Statement is a solitary liver tumor ≤ 5 cm [4]. Recently, using LLR for large hemangiomas has been reported [8, 9]. The difficulty with using LLR for giant hemangiomas depends on tumor size, tumor location, growth type, and adhesion area between the hemangioma and liver.

Because hemangiomas have high vascularity and a soft texture, they have the potential to bleed easily. Controlling the inflow into the resection area is essential, and preoperative TAE is an effective modality to reduce operative bleeding related with the hepatic arterial supply [8, 10–13]. Zhang et al also reported that infrahepatic inferior vena cava clamping with the Pringle maneuver was safe and effective for controlling bleeding [9]. In our case, preoperative TAE and the Pringle maneuver were applied. The operative time (288 min) and blood loss (200 mL) were normal and comparable to that previously reported [9], and TAE would be somewhat helpful to lessen bleeding.

Preoperative simulation using 3D image reconstruction may enhance surgical planning and navigation during LLR [14]. It can measure the remnant liver volume and visualize hepatic anatomy and tumor localization. In LLR for giant hemangiomas, handling several laparoscopic instruments within a limited workspace is key to completing the operation. Our patient’s giant hemangioma showed extrahepatic growth, and the intraabdominal workspace was severely restricted. The simulated laparoscopic view from the left upper abdomen was optimal for safely handling the various surgical instruments, and 3D simulation was very informative and helpful for port placement. Though, the change of positional relationship among the organs and costal bones under pneumoperitoneum could not be considered in this simulation tool. The definitive decision of port placement should be conducted intraoperatively. This is the first report in which preoperative 3D simulation of port site placement, and the laparoscopic view was utilized in LLR for giant liver hemangioma. The parenchymal transection plane image, the laparoscopic view, and the port placement were simulated preoperatively, and the operation was successfully completed the same way it was simulated.

In conclusion, our report suggests that pure laparoscopic resection could be safely applied to resect giant liver hemangiomas with extrahepatic growth. The preoperative 3D simulation of the port placement and the laparoscopic view might be helpful when intraabdominal workspace is restricted.

## Abbreviations

3D: Three-dimensional
CT: Computed tomography
LLR: Laparoscopic liver resections
TAE: Transcatheter arterial embolization
